# Supplementary material for: Association between dental scaling and metabolic syndrome and lifestyle
Source: PLoS One. 2024 Apr 4;19(4):e0297545. doi: 10.1371/journal.pone.0297545 (PMC10994476; doi:10.1371/journal.pone.0297545)
Supplement: S1 Appendix — (DOCX) [file pone.0297545.s001.docx]

Appendix Table 1. Multiple logistic regression analysis according to metabolic syndrome

|  | | N (%) | OR (95% C.I) | | |
| --- | --- | --- | --- | --- | --- |
|  |  |  | Model 1 | Model 2 | Model 3 |
| Abdominal obesity | No | 85,498 (20.32) | 1 (Ref.) | 1 (Ref.) | 1 (Ref.) |
|  | Yes | 20,468 (16.63) | 0.782 (0.769, 0.795) | 0.852 (0.838, 0.867) | 0.932 (0.912, 0.952) |
|  | P-value |  | <.0001 | <.0001 | <.0001 |
| Lowe HDL -C | No | 74,966 (20.48) | 1 (Ref.) | 1 (Ref.) | 1 (Ref.) |
|  | Yes | 31,000 (17.44) | 0.820 (0.808, 0.832) | 0.949 (0.935, 0.964) | 0.984 (0.968, 0.999) |
|  | P-value |  | <.0001 | <.0001 | 0.0422 |
| High triglycerides | No | 69,900 (20.74) | 1 (Ref.) | 1 (Ref.) | 1 (Ref.) |
|  | Yes | 36,066 (17.44) | 0.807 (0.796, 0.819) | 0.912 (0.899, 0.926) | 0.963 (0.948, 0.978) |
|  | P-value |  | <.0001 | <.0001 | <.0001 |
| High blood pressure | No | 66,969 (22.12) | 1 (Ref.) | 1 (Ref.) | 1 (Ref.) |
|  | Yes | 38,997 (16.18) | 0.680 (0.671, 0.689) | 0.797 (0.785, 0.808) | 0.824 (0.812, 0.837) |
|  | P-value |  | <.0001 | <.0001 | <.0001 |
| Hyperglycemia | No | 72,878 (21.02) | 1 (Ref.) | 1 (Ref.) | 1 (Ref.) |
|  | Yes | 33,088 (16.79) | 0.758 (0.747, 0.769) | 0.848 (0.836, 0.861) | 0.877 (0.863, 0.890) |
|  | P-value |  | <.0001 | <.0001 | <.0001 |
| Metabolic Syndrome | No | 79,372 (21.06) | 1 (Ref.) | 1 (Ref.) | 1 (Ref.) |
|  | Yes | 26,594 (15.94) | 0.711 (0.700, 0.722) | 0.842 (0.828, 0.855) | 0.894 (0.878, 0.909) |
|  | P-value |  | <.0001 | <.0001 | <.0001 |
| Metabolic syndrome Component | 0 | 34,478 (23.87) | 1 (Ref.) | 1 (Ref.) | 1 (Ref.) |
|  | 1 | 25,901 (20.26) | 0.810 (0.796, 0.825) | 0.866 (0.850, 0.883) | 0.885 (0.869, 0.902) |
|  | 2 | 18,993 (18.14) | 0.707 (0.693, 0.721) | 0.799 (0.782, 0.815) | 0.832 (0.814, 0.850) |
|  | 3 | 14,038 (16.83) | 0.645 (0.631, 0.660) | 0.768 (0.750, 0.786) | 0.810 (0.790, 0.830) |
|  | 4 | 9,162 (15.53) | 0.586 (0.571, 0.601) | 0.740 (0.721, 0.760) | 0.788 (0.766, 0.811) |
|  | 5 | 3,394 (13.88) | 0.514 (0.495, 0.534) | 0.678 (0.652, 0.705) | 0.741 (0.710, 0.773) |
|  | P-value |  | <.0001 | <.0001 | <.0001 |

Model 1 was not adjusted.

Model 2 was adjusted for age and sex.

Model 3 was adjusted for age, sex, income level, BMI, smoking, alcohol consumption and regular exercise.

P< 0.05 is statistically significant.

Table 2. Multiple logistic regression analysis according to lifestyle

|  | | N (%) | OR (95% C.I) | | | |
| --- | --- | --- | --- | --- | --- | --- |
|  |  |  | Model 1 | Model 2 | Model 3 | Model 4 |
| Smoking | Non | 67,852 (20) | 1 (Ref.) | 1 (Ref.) | 1 (Ref.) | 1 (Ref.) |
|  | Ex | 17,808 (20.93) | 1.059 (1.039, 1.079) | 1.096 (1.072, 1.121) | 1.095 (1.071, 1.120) | 1.100 (1.075, 1.125) |
|  | Current | 20,306 (16.99) | 0.819 (0.805, 0.833) | 0.754 (0.739, 0.770) | 0.786 (0.769, 0.803) | 0.784 (0.767, 0.801) |
|  | P-value |  | < 0.0001 | < 0.0001 | < 0.0001 | < 0.0001 |
| Drinking | Non | 52,953 (18.63) | 1 (Ref.) | 1 (Ref.) | 1 (Ref.) | 1 (Ref.) |
|  | Mild | 45,839 (20.98) | 1.160 (1.143, 1.176) | 1.042 (1.026, 1.058) | 1.043 (1.027, 1.059) | 1.042 (1.026, 1.058) |
|  | Heavy | 7,174 (17.49) | 0.926 (0.901, 0.951) | 0.859 (0.835, 0.884) | 0.916 (0.889, 0.943) | 0.928 (0.901, 0.956) |
|  | P-value |  | < 0.0001 | < 0.0001 | < 0.0001 | < 0.0001 |
| Regular exercise | No | 83,204 (19.02) | 1 (Ref.) | 1 (Ref.) | 1 (Ref.) | 1 (Ret.) |
|  | Yes | 22,762 (21.43) | 1.161 (1.142, 1.181) | 1.195 (1.175, 1.215) | 1.163 (1.144, 1.183) | 1.164 (1.144, 1.183) |
|  | P-value |  | < 0.0001 | < 0.0001 | < 0.0001 | < 0.0001 |
| Unhealthy Lifestyle Score | 0 | 18,233 (22.49) | 1 (Ref.) | 1 (Ref.) | 1 (Ref.) | 1 (Ref.) |
|  | 1 | 67,765 (19.6) | 0.840 (0.825, 0.856) | 0.817 (0.802, 0.833) | 0.828 (0.813, 0.844) | 0.828 (0.812, 0.844) |
|  | 2 | 16,985 (17.24) | 0.718 (0.701, 0.735) | 0.641 (0.626, 0.657) | 0.665 (0.649, 0.682) | 0.666 (0.638, 0.695) |
|  | 3 | 2,983 (16.19) | 0.664 (0.647, 0.680) | 0.580 (0.555, 0.606) | 0.607 (0.581, 0.634) | 0.612 (0.586, 0.640) |
|  | P-value |  | < 0.0001 | < 0.0001 | < 0.0001 | < 0.0001 |

Model 1 was not adjusted.

Model 2 was adjusted for age and sex.

Model 3 was adjusted for age, sex, income level and BMI.

Model 4 was adjusted for age, sex, income level, BMI, diabetes, hypertension and dyslipidemia.

P< 0.05 is statistically significant.
