# Supplementary material for: Association between dental scaling and metabolic syndrome and lifestyle
Source: PLoS One. 2024 Apr 4;19(4):e0297545. doi: 10.1371/journal.pone.0297545 (PMC10994476; doi:10.1371/journal.pone.0297545)
Supplement: Attachment — Submitted filename: response to reviewer comments_ Plos One.docx [file pone.0297545.s003.docx]

Dear reviewers and editors

I appreciate your kind advice about this manuscript. I did my best to correct the flaws. The corrected items were marked as yellow color in the manuscript. Please let me know if there are any more revisions.

Sincerely yours,

Comments from the Editors and Reviewers:

5. Review Comments to the Author

Please use the space provided to explain your answers to the questions above. You may also include additional comments for the author, including concerns about dual publication, research ethics, or publication ethics. (Please upload your review as an attachment if it exceeds 20,000 characters)

**Reviewer #1**: The paper has the potential to enrich the literature by promoting a discussion about whether preventive scaling would indeed have any impact on indicators of metabolic syndrome in a population-based study. I recommend that the authors reconsider the theoretical concept underlying the analyses and reorganize the presentation of the paper. In an epidemiological study like this, clarifying the outcomes and exposures according to your aims is crucial. In a deep reflection, I suggest revisiting some assumptions still unclear in the literature. There is no consensus on the role of periodontitis in chronic disease causation. Studies show that, most likely, both share the same risk factors, such as an unhealthy diet. In this sense, preventive scaling would have little impact on periodontitis and even less on other metabolic risks.

- I appreciate your kind review and comments. Recent Cochrane review study reported that moderate-certainly evidence that periodontal therapy is helpful in reduction of HbA1c in diabetic patients (reference No.27). We wanted to investigate the extent to which people with metabolic syndrome or bad lifestyle performed scaling, a basic periodontal disease management program and the association between preventive scaling and metabolic syndrome or lifestyle in a large population. We have added these statements to the 'Introduction' section.

**Reviewer #2:** The study uses a national database to report the associations of metabolic syndrome and unhealthy lifestyle (as exposures) and preventive scaling (as the outcome). The topic is clinically relevant as there is a need to identify the utilization rates of preventive dental care in such individuals at risk of periodontal disease. However, there are major methodological issues that need to be addressed before it can be considered for publication. Below are a few points for the consideration of the authors:

1. The statistical plan addresses the main objectives of the study using too many multivariable models and is rather confusing to follow. For instance, it is difficult to understand what Table 2 adds in the overall analysis, besides identifying the significant covariates associated with the outcome. If that is the case, the univariate analysis should suffice. As such, the multivariable analysis in Table 2 only adds to the ambiguity, for example, dyslipidemic individuals are shown to be at higher odds of preventive scaling which is in contrast to what has been reported in Table 3, where individuals with high triglycerides and low HDL-C levels are at lower odds of preventive scaling.

- I appreciate your kind review and comments. Following your advice, the multivariate analysis results were omitted from Table 2.

2. In Table 3, metabolic syndrome is categorized as a binary variable (yes/no), ordinal variable (with its five components together), and also as individual components tested separately with the outcome. The rationale of having so many definitions of metabolic syndrome to test this association is unclear. Also, the rationale behind having a stepwise inclusion of confounders as Models 1,2,3 needs to be clearly mentioned if it adds anything to the overall results, in contrast to reporting only model 3 which includes all the potential confounders.

- Inclusion of confounders of each Models were explained more clearly in ‘Method-Statistical analysis’ section. Table 3 was presented as forest plots and have been modified to present only the final model in Results section. I've added that table to the supplementary for readers who might be wondering how the forest plot was created.

3. Similarly, Table 4 also reports multiple models as well as definitions of exposure to test the association which either needs to be justified or needs to be trimmed down substantially. More so, some of the models are very perplexing. For example, Model 3 in Table 4 adjusts for smoking, drinking and regular exercise while testing the association of the same exposures with the outcome. The authors would need to re-organize the tables, to highlight main findings they believe helps to test their study objective.

- Inclusion of confounders of each Models were explained more clearly in ‘Method-Statistical analysis’ section. Table 4 was presented as forest plots and have been modified to present only the final model in Results section. I've added that table to the supplementary for readers who might be wondering how the forest plot was created.

4. Minor points:
 i) The current Table 1 is unclear and confusing. In Table 1, it would be more informative if the comparative proportion of individuals is reported by row and not by column. For instance, how many proportions of males had/did not undergo preventive scaling, etc. Also, Table 1 needs to be re-organized into categorical variables, which is reported as N (%) and continuous variables, reported as Mean (SD).

- It was corrected. Categorical variables were described as frequencies (%) and continuous variables were described as means ± standard deviation (SD) in Table 1. ‘how many proportions of males had/did not undergo preventive scaling, etc.’ are shown in Event n (%) in Table 2.

ii) In Tables 1 and 2, remove the term “baseline” as this is a cross-sectional data with no baseline and follow-ups.

- It was corrected. I changed the term ‘baseline’ to ‘clinical’.

iii) In the Discussion, the authors need to discuss the potential role of dental visits for more complex procedures (such as surgical/non-surgical periodontal therapy) in those with metabolic syndrome and/or unhealthy lifestyle, which would confound the absence of codes for preventive scaling in this group of people.

- Preventive dental scaling and therapeutic dental scaling are managed under different prescription codes. Because this study focused on preventive care, data from people who received only therapeutic scaling without preventive scaling were not included. It is a limitation of this study. We have added these statements to the 'Limitation and strength' section.

iv) Also, potential explanations for the associations observed in the study needs further corroboration with evidence from literature.

- References added in ‘Discussion’ section.

**Reviewer #3:** This is an interesting manuscript on the association between metabolic syndrome, unhealthy lifestyle habits and preventive dental scaling using a huge dataset from Korea. As results, authors showed that these characteristics are interconnected and clustered in the sample, and that dose-response relationships with dental scaling for preventive purposes occur.

Introduction:
*There are some sentences that called my attention:
"Calculus, which is not removed by routine oral hygiene practices such as brushing, is the CAUSE of periodontitis along with the bacterial plaque that covers it."
"Periodontitis bacteria in dental calculus and bacterial plaque locally DESTROY not only the soft tissues around the teeth but also the alveolar bone, resulting in tooth loss."
"Periodontal pathogens, which CAUSE periodontal disease..."
In my opinion, these sentences should be accurately reviewed. Current knowledge shows that periodontitis causation are not so simply as stated.
*State study's hypotheses.

- I appreciate your kind review and comments. The causes of periodontitis were added in ‘Discussion’ section.

Methods:
*It would be interesting to see how variables were hierarchically organized. Consider to use a flowchart for that.

- Flowchart of study population and design was inserted as Fig 1. How variables were hierarchically organized were explained in ‘Method-Statistical analysis’ section.

Results:
*I am not convinced on the way how authors reported their results. In table 1, for instance, authors showed all independent variables assessed, and described these as "baseline characteristics". In table 2, they also called "baseline characteristics" but selected some to report. Additionally, tables 3 and 4 showed some data also previously presented in table 2 (as the effect measures are the same). For instance, high blood pressure/hypertension, drinking, smoking and regular exercise.
My point is that these tables can be simplified and data could be accommodated in two tables (one for unhealthy lifestyle and another for metabolic syndrome).
*As authors described dose-response relationships, I would suggest to remove such findings from tables and present the odds ratios graphically.

- Inclusion of confounders of each Models were explained more clearly in ‘Method-Statistical analysis’ section. We changed the term ‘baseline’ to ‘clinical’ in Table 1 and 2. Table 2 was corrected more simply. Table 3 and 4 were presented as forest plots and have been modified to present only the final model in Results section.

Discussion:
*Authors centered the discussion (and introduction as well) on the microbiological path that links exposures and periodontitis. What about the inflammatory pathway?

- The etiology of periodontitis was added in ‘Discussion’ section.

*Indeed, what about behavioral pathways? Authors described that metabolic syndrome components, unhealthy lifestyle habits and lack of preventive oral care are clustered in the same individuals. What health professionals should do in this respect?

- The people with metabolic syndrome and the people with unhealthy lifestyle is not the same cluster. The people with metabolic syndrome may or may have unhealthy lifestyles. The people with unhealthy lifestyle may or may have metabolic syndrome. Medical doctors might encourage a patient with metabolic syndrome to visit dental clinics. Clinicians need to help heavy drinkers reduce the amount and frequency of alcohol consumption, not only for their systemic health, but also for their oral health. These were added to the ‘Discussion’ section.

*Language revision would be useful to avoid some redundancies such as "treatment to treat".

- It was corrected.
