# Supplementary material for: Association between dental scaling and metabolic syndrome and lifestyle
Source: PLoS One. 2024 Apr 4;19(4):e0297545. doi: 10.1371/journal.pone.0297545 (PMC10994476; doi:10.1371/journal.pone.0297545)
Supplement: Attachment — Submitted filename: response to reviewer comments 2nd.docx [file pone.0297545.s004.docx]

Dear reviewers and editors

I appreciate your kind advice about this manuscript. I did my best to correct the flaws. The corrected items were marked as yellow color in the manuscript. Please let me know if there are any more revisions.

Sincerely yours,

Comments from the Editors and Reviewers:

6. Review Comments to the Author

Please use the space provided to explain your answers to the questions above. You may also include additional comments for the author, including concerns about dual publication, research ethics, or publication ethics. (Please upload your review as an attachment if it exceeds 20,000 characters)

**Reviewer #1:** It must be acknowledged that the authors have made progress in the construction of the text and have greatly improved the way it is presented. Nevertheless, I suggest clarifying some of the study's important points.

Abstract/Results
The sentence: "Multiple logistic regression analysis showed that after adjustment for age, sex, income, body mass index (BMI), smoking, drinking and regular exercise, the Odds ratios (OR) and 95% confidence intervals (CI) of" is part of the methods and not the results.

- I appreciate your kind review and comments. It was corrected.

Introduction
The authors approached a quite obvious pathway in which periodontal bacteria would cause cardiometabolic diseases. But right after that, the authors say sentences like “It has been reported that people with metabolic syndrome are twice as likely to develop periodontitis than those without it” demonstrating that perhaps periodontitis is caused by these diseases. It seems more plausible to believe that both periodontitis and cardiometabolic diseases have common causes such as smoking, alcohol, diet, etc. As there is no consensus on this, this should be expressed in the text. In the last two hypotheses, the low demand for dental scaling would be a clear demonstration that individuals have an unhealthy lifestyle, both in terms of general health and oral health.

- The content of this section was modified, and it was added that metabolic syndrome and periodontitis have a bi-directional relationship.

Methods
The outcome variable preventive dental scaling needs to be better defined. Firstly, it needs to be stated in the text that this is the outcome studied, for example, "The outcome variable in this study was the preventive dental scaling...". Subsequently, the authors do not make it clear how this very important variable in the study was measured. Apparently, it was considered as a binary (y/n) variable, but this is not stated in the text. Furthermore, I have some doubts: Was it considered as “at least one visit”? Was it considered as the total number of visits (continuous)?

- Following your advice, it was explained more clearly in Method section.

Were participants who were referred directly for therapeutic dental scaling excluded from the analysis? This may seem obvious, but it is important to clarify in the text, since the participant may not have carried out the preventive phase because their periodontal health had already deteriorated and therefore the preventive approach would no longer make sense in this situation.

- It was explained more clearly in Methods section.

Understanding that the frequency of visits for periodontal treatment is strongly linked to a healthy lifestyle, performing models adjusted for the number of medical visits (or another healthy lifestyle indicators) is necessary to help control confounding bias.

- Unfortunately, we are unable to further analyze the data as it has expired and the data has been deleted. We will take your advice into consideration for future research.

Discussion
The sentence: “Periodontitis is a chronic inflammatory infectious disease” does not make sense based on the WHO division of diseases, i.e. chronic (chronic inflammatory) or infectious diseases. From the relationship between periodontitis and other chronic diseases, it seems to be more of a chronic disease than an infectious disease, which is confirmed in the first 6 lines of the third paragraph of the discussion. Bear in mind that the relationship between periodontitis and other diseases is not clear from the literature.

- The definition of periodontitis has been revised based on guidance from the European Federation of Periodontology (EFP). Reference has been added to the ‘Discussion’ section.

P17: “The present study also investigated how many times the people with bad lifestyle habits were dental scaling.” The authors do not mention in the methodology that the outcome was considered to be the number of times the participant underwent (continuous) treatment. If dental scaling was considered as being binary, this sentence should be rewritten, as what was assessed was the probability/odds of having or not having undergone dental scaling.

- It was corrected. A description of the outcome variable has been added to 'Methods' section.

**Reviewer #3:** The authors are commended for their thoughtful review of the manuscript. Few issues remain to be addressed in the manuscript:

Introduction:
*There are STILL some sentences that called my attention:
"Calculus, which is not removed by routine oral hygiene practices such as brushing, is the CAUSE of periodontitis along with the bacterial plaque that covers it." - you may consider dental plaque (dysbiosis) as a causal component of periodontitis, however, to the best of my knowledge, there is no evidence to support such a role for calculus;
"Periodontitis bacteria in dental calculus and bacterial plaque locally DESTROY not only the soft tissues around the teeth but also the alveolar bone, resulting in tooth loss." – periodonto-pathogens are responsible for a small part of tissue destruction. The major contributor is host immune response;
"Periodontal pathogens, which CAUSE periodontal disease..." - current knowledge shows that periodontitis causation are not so simply as stated. Please, follow current definitions of periodontitis etiology.

- I appreciate your kind review and comments. We've revised the Introduction and Discussion sections to reflect the recent articles. The definition of periodontitis followed the guideline from the European Federation of Periodontology (EFP).

Methods:
*Authors described their modeling strategy. However, there is no clear explanation on why such arrangement was done. For instance, why "unhealthy lifestyle" variables were combined with the socioeconomic ones for metabolic syndrome, and another block (exclusive for comorbidities) was used when unhealthy lifestyle was the outcome? Additionally, BMI is not a socioeconomic variable.

- The variables in the final model were age, sex, BMI, income level, smoking, alcohol consumption, regular exercise, Diabetes mellitus, Hypertension, and Dyslipidemia (variables in Table 2). The variables in the final model were grouped into groups (demographics, body composition, socio-economics, lifestyle, comorbidities). The variables were added to the model progressively from non-adjusted to fully adjusted. A description of modeling strategy has been added to 'Methods' section and Fig 1 was corrected.

*Was there any criteria for variable retention? Or all variables were included?

- We included all variables that were available in the data we obtained.

Discussion:
*Language revision would still be useful.
"(...) preventive treatment to prevent (...)"
"(...) people with bad lifestyle habits were dental scaling."

- It was corrected.
