# Supplementary material for: Association between dental scaling and metabolic syndrome and lifestyle
Source: PLoS One. 2024 Apr 4;19(4):e0297545. doi: 10.1371/journal.pone.0297545 (PMC10994476; doi:10.1371/journal.pone.0297545)
Supplement: Attachment — Submitted filename: response to reviewer comments 3rd.docx [file pone.0297545.s005.docx]

Dear reviewers and editors

I appreciate your kind advice about this manuscript. I did my best to correct the flaws. The corrected items were marked as yellow color in the manuscript. Please let me know if there are any more revisions.

Sincerely yours,

Thank you for submitting your revised manuscript. I read with interest the corrections you have made. While the methodology became clearer in this version, the Introduction section, more specifically, the concepts of periodontitis causation and its relationship with systemic diseases, should be carefully revised.

While a dysbiotic biofilm has been associated with periodontitis, it has been known that plaque deposits account for approx. 20% of periodontitis causation, while the remaining 80% relate to the host's ability to deal with the inflammatory process. You can find more details elsewhere (1). On a similar note, the authors assume periodontitis as a cause for several systemic diseases, such as endothelial dysfunction, myocardial infarction, and diabetes, among others. While studies have shown associations between these conditions, causation still neeeds to be established (2). In addition, referee #3 mentioned that a potential link between periodontitis and systemic diseases relies on their shared risk factors, such as smoking and diet, among others. This has not been included in the manuscript, and, given the aim of this study - to investigate lifestyle factors in addition to MetS - this should be included and discussed (3).

 Once you revise your theoretical background accordingly, a final editorial decision can be made.

- I appreciate your kind review and comments. Following your advice, we've revised the Introduction section to reflect the recent articles.
